# Chest Pain and Hypotension in a Dialysis Patient

**DOI:** 10.34067/KID.0000000000000271

**Published:** 2024-01-25

**Authors:** A. Onome Ehinmisan, Raphael Rosen

**Affiliations:** 1 Stamford Hospital, Stamford, Connecticut; 2 Division of Nephrology, Department of Medicine, Stamford Health Medical Group, Stamford, Connecticut

**Keywords:** cardiovascular disease, chronic hemodialysis, dialysis

## Abstract

**Figure:**
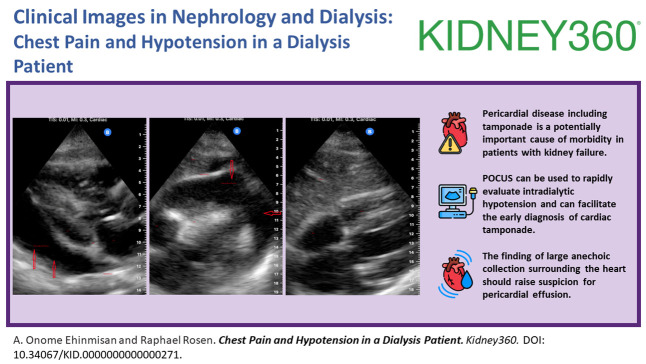

## Case Description

**Figure 1:**
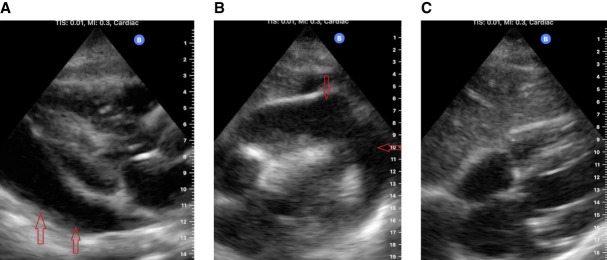
**Ultrasound revealed a large circumferential pericardial effusion causing right ventricular diastolic collapse.** (A) Parasternal long axis view. Red arrows indicate pericardial effusion. (B) Subcostal view. Red arrows indicate pericardial effusion. (C) Subcostal view showing resolution of pericardial effusion. LA, left atrium; LV, left ventricle; MV, mitral valve; RA, right atrium; RV, right ventricle.

A 63-year-old man with ESKD presented to the dialysis unit with chest discomfort and hypotension. His chest pain began after his last hemodialysis treatment 2 days prior. His baseline BP was generally elevated at the initiation of dialysis treatments but upon presentation to dialysis was 93/59 mm Hg despite being 2 kg above his dry weight. He was noted to be less verbal and somewhat confused. Dialysis was initiated. but after 1 hour his systolic BP decreased to 60 mm Hg and he became somnolent. Hypotension persisted despite administration of 1 L of normal saline, and dialysis was terminated. The nephrologist evaluated the patient at the dialysis clinic before emergency medical services arrival and, cardiac point of care ultrasound (POCUS) was performed. A large circumferential anechoic collection was noted around the heart, with right ventricular diastolic collapse (Figure 1, A and B). A diagnosis of cardiac tamponade was made.

The emergency physicians were alerted before the patient's arrival of the likely diagnosis, and arrangements were made for an immediate interventional cardiology evaluation. Within 30 minutes of arrival, 600 cc of bloody fluid was drained from the pericardium, and a pericardial drain was placed. Shock resolved immediately. Fluid studies revealed no autoimmune disease, infection, tuberculosis, or malignancy. He was diagnosed with dialysis-associated pericardial effusion. He was dialyzed daily for 7 days, and heparin was not used. Serial cardiac POCUS (performed in the dialysis unit) revealed no reaccumulation of hemorrhage (Figure 1C).

## Discussion

Hypotension is a fairly common occurrence during dialysis treatments,^1^ often caused by ultrafiltration. The most common interventions include slowing the rate of ultrafiltration and administration of fluid boluses. In this case, the patient's hypotension preceded initiation of hemodialysis but was worsened by ultrafiltration and failed to resolve with a fluid bolus. The lack of response to a fluid bolus suggests a nonvolume-related cause of hypotension. Both septic shock and cardiogenic shock from ischemia are fairly common in patients with kidney failure^2^ and would have been higher on the differential than tamponade, were it not for the prompt use of POCUS. Although Beck triad of hypotension, jugular venous distention, and distant heart sounds are diagnostic of tamponade, ultrasound is an essential to the rapid evaluation of these patients.^3^

The availability of ultrasound and ultrasound-trained physician in the dialysis unit facilitated rapid diagnosis and treatment of this patient and avoided potential morbidity.^4^ Although pericarditis and pericardial disease often causes electrocardiographic changes, in uremic pericarditis and dialysis-associated pericarditis, electrocardiographic changes are often absent,^3^ accentuating the utility of cardiac ultrasound in making this diagnosis.

## Teaching Points

- Pericardial disease including tamponade is a potentially important cause of morbidity in patients with kidney failure.
- POCUS can be used to rapidly evaluate intradialytic hypotension and can facilitate the early diagnosis of cardiac tamponade.
- The finding of large anechoic collection surrounding the heart should raise suspicion for pericardial effusion.
